# Poly(*p*-phenylenevinylene) nanoparticles modified with antiEGFRvIII for specific glioblastoma therapy

**DOI:** 10.1038/s41598-021-83931-5

**Published:** 2021-02-24

**Authors:** Yuchao Liang, Zelin Li, Huanxiang Yuan, Lei Wang, Li-Hua Gao

**Affiliations:** 1 Neurosurgery Department, Beijing Tian Tan Hospital, Capital Medical University, Beijing, 100070 People’s Republic of China; 2 Department of Chemistry, College of Chemistry and Materials Engineering, Beijing Technology and Business University, Beijing, 100048 People’s Republic of China

**Keywords:** Cancer immunotherapy, Chemotherapy, Targeted therapies

## Abstract

Glioblastoma is the most common primary brain cancer and it is nearly impossible to remove the entire tumor with surgery or a single drug. EGFRvIII is the most frequent genetic change associated with glioblastoma, so EGFRvIII-based targeting therapies provide promise for treating glioblastoma. Herein, poly[2-methoxy-5-(2′-ethylhexyloxy)-*p*-phenylenevinylene] (PPV) was used as the core to prepare a conjugated polymer nanoparticle (PPVN) modified with anti-EGFRvIII (PPVN-A) that exhibited high ROS generation ability under white light irradiation. PPVN-A could target EGFRvIII-overexpressed tumor cells and damaged more than 90% of tumor cells with the light illumination while PPVN without modification exhibited no obvious cytotoxicity toward these cells under the same condition. Thus, the photodynamic treatment of glioblastoma cells using PPVN-A could be achieved, indicating the potential of anti-EGFRvIII-modified nanoparticles as a therapeutic material for treating glioblastoma in clinic.

## Introduction

Glioblastoma (GBM), which is one type of malignant central nervous system (CNS) tumors^1^, is the most common primary brain cancer, and there are almost 12,000 cases confirmed in the United States every year. WHO grades include gliomas-grade II, III and IV, and grade IV is the most malignant^2^. The glioblastoma (GBM) is WHO-grade IV brain tumors, indicating they contain the most aggressive cells. Importantly, glioblastoma could develop branches to diffuse into different parts of the brain quickly and surgery is nearly able to remove these branches. Due to tumor heterogeneity, different cells in a single tumor comprise numerous different types of molecular markers, so that a drug works for certain cells but is of no avail to the entire tumor. Therefore, it is particularly important to find a new treatment for GBM. As the understanding of the molecular mechanisms and signaling pathways of GBM development has gradually deepened, the treatment of specific molecular markers has become the frontier and trend of GBM research.

It is found that amplification of the epidermal growth factor receptor (EGFR) gene is the most frequent genetic change associated with GBM, and GBM exhibits that amplified EGFR frequently overexpresses the receptor variant III (EGFRvIII)^3^, which implies the critical influence of EGFRvIII toward increased proliferation of glioma cells^4^. It is proved that the survival time of EGFRvIII-positive GBM patients was significantly shortened^5^. Therefore, EGFRvIII plays crucial role in targeted therapy in EGFRvIII-amplified GBM^6,7^. At present clinical research, there are plenty of targeted therapeutic strategies for GBM based on EGFRvIII, such as EGFRvIII small molecule inhibitors, EGFRvIII monoclonal antibodies, and anti-EGFRvIII vaccines, etc^8–12^. However, its final clinical trial results are not satisfactory. The efficiency of blood–brain barrier penetration, heterogeneity in GBM, the presence of compensatory signaling pathways in tumor cells and drug resistance are the main reasons^13^. Therefore, it is urgent to develop and design new therapeutic drugs and treatment models for EGFRvIII.

Photodynamic therapy (PDT), as a non-invasive therapeutic manner, has attracted increasingly wide attention because of its low side-effects and high efficiency^14–16^. Conjugated polymers with characteristic delocalized π-electronic backbones have drawn increasing research interests due to their strong light absorption capacity, high photostability and low cytotoxicity^17–23^. To date, a great number of conjugated polymers have been designed and synthesized to be used as photosensitizers in PDT^24–29^. However, there are few reports on the research of targeted photodynamic therapy based on conjugated polymer and EGFRvIII for treating glioblastoma. Here, Anti-EGFRvIII modified conjugated polymer nanoparticles (PPVN-A) that have the characteristics of good water dispersion, low toxicity and high targeting efficiency, were designed and prepared.

Therefore, by constructing targeted conjugated polymer nanoparticles, selective identification of EGFRvIII is realized to demonstrate effects on fluorescence imaging and photodynamic therapy in GBM. A new direction is expected to be provided for the patients with GBM in diagnosis and treatment.

## Results and discussion

**Scheme 1:**
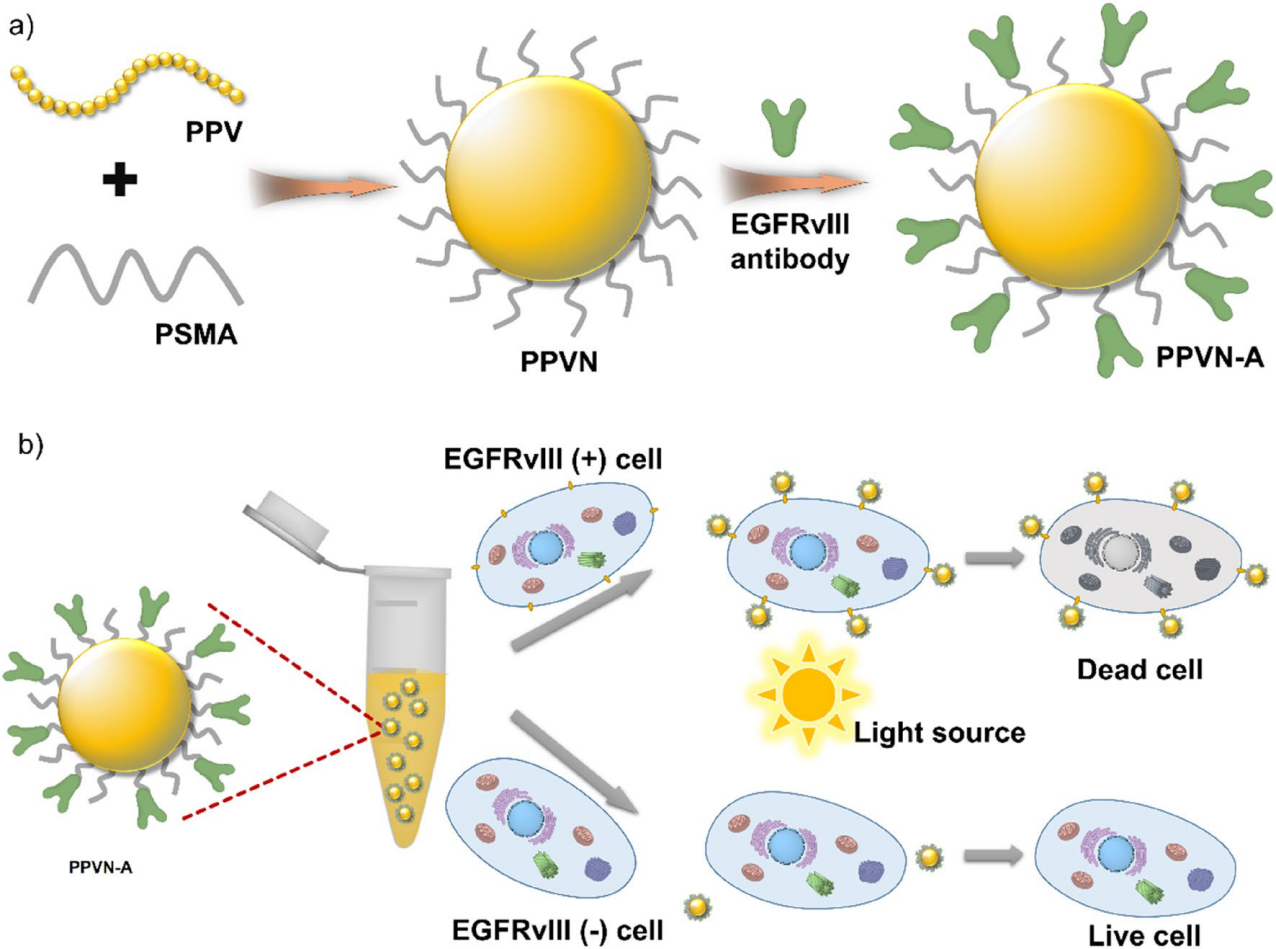
(**a**) Illustrated preparation of antibody-modified nanoparticles (PPVN-A). (**b**) Schematic illustration of PPVN-A for targeted photodynamic therapy.

**Figure 1:**
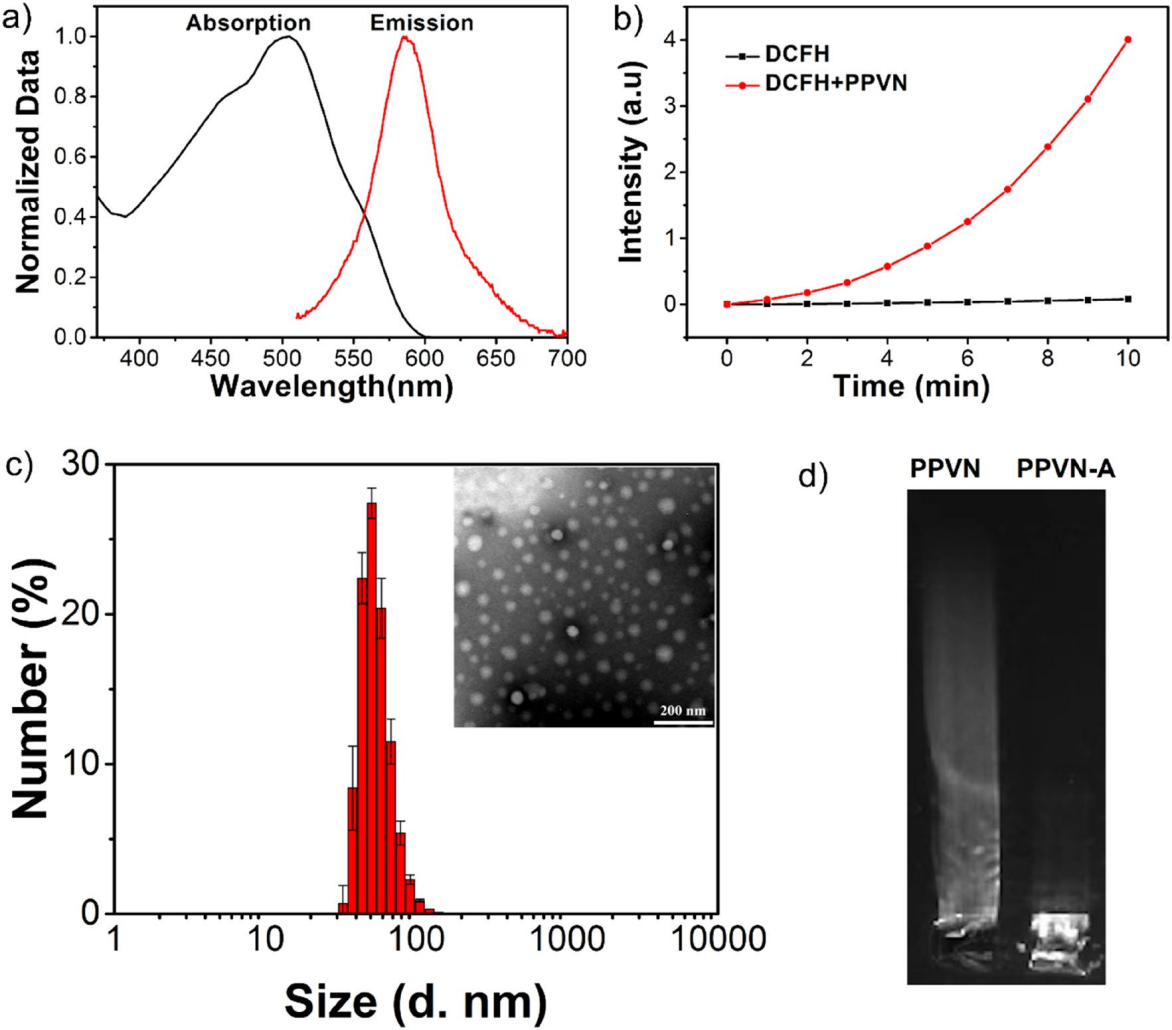
(**a**) Absorption and emission spectra of PPVN. (**b**) Fluorescence intensity of DCFH in the absence and presence of PPVN. (**c**) The dynamic size distribution TEM image of PPVN. (**d**) Gel electrophoresis of PPVN and PPVN-A.

The carboxyl functionalized PPVN were prepared using a nanoprecipitation method in the presence of poly(styrene-co-maleic anhydride) (PSMA) via hydrophobic interactions. The carboxyl groups on the surface of PPVN could be linked with antibodies which are targeted toward receptor-overexpressed tumor cells. (Scheme 1a) The absorption and fluorescence emission spectra indicate a maximum absorption of 505 nm and an emission peak of 585 nm with the excitation of 505 nm, demonstrating a big Stokes shift of 80 nm which could avoid self-absorption of the materials in bio-imaging applications. (Fig. 1a) PPVN display high sensitization ability of oxygen to generate reactive oxygen species (ROS). (Fig. 1b) 2,7-Dichlorofluorescein diacetate (DCFH-DA) was utilized to detect the production of ROS in the presence of PPVN upon irradiation of white light. DCFH-DA can be hydrolyzed to dichlorodihydrofluorescein (DCFH) under treatment with NaOH solution, and subsequently oxidized to highly fluorescent 2,7-dichlorofluorescein (DCF) in the presence of ROS. As seen from Fig. 1b, the fluorescence of DCF gradually grows with the irradiation time increasing after the addition of PPVN, demonstrating the formation of ROS. These results imply that PPVN have great potentials to be bioimaging and photodynamic therapeutic materials in biomedical fields.

The epidermal growth factor receptor variant III (EGFRvIII) overexpressed Human Glioma Cell line (LN229) and EGFRvIII-negative LN229 cells were established. EGF Receptor vIII Rabbit mAb (anti-EGFRvIII) was applied to recognize endogenous and transfected levels of EGF receptor vIII protein overexpressed LN229. The carboxyl groups on PPVN react with the amino groups on anti-EGFRvIII to afford PPVN-A which specifically targeted EGFRvIII overexpressed LN229 cells but could not interact with EGFRvIII-negative LN229 cells. (Scheme 1b) Under the irradiation of white light, EGFRvIII overexpressed LN229 cells which bind PPVN-A were damaged by the reactive oxygen species (ROS) because PPV could sensitize oxygen to generate ROS.

To endow PPVN targeting function toward gliomas cells, anti-EGFRvIII that is the most important antibody in the EGFRvIII related therapy is linked to the surface of PPVN via the reaction of carboxyl and amino groups. Electrophoresis, zeta potential and dynamic light scattering measurements were performed to verify the successful covalent attachment between PPVN and anti-EGFRvIII. PPVN show spherical shape with an average diameter of ~ 50 nm. (Fig. 1c) Gel electrophoresis characterization suggested that anti-EGFRvIII modified PPVN-A ran a little slower than PPVN due to the reduced negative charge on the surface with successful covalent linkage between anti-EGFRvIII and PPVN. (Fig. 1d) Zeta potential and size measurement indicates that nanoparticles linked anti-EGFRvIII (PPVN-A) becomes less negative and bigger after modification of anti-EGFRvIII than unmodified nanoparticles (PPVN), which is consistent with the gel electrophoresis result, further suggesting the successful reaction of carboxyl functional groups on the surface of PPVN with the amino moieties of anti-EGFRvIII. (Table S1) Therefore, the series of characterizations suggest the amide formation between PPVN and anti-EGFRvIII.

**Figure 2:**
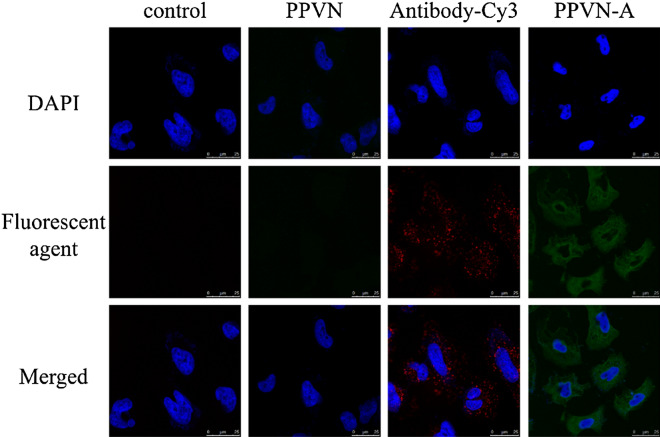
The CLSM images of EGFRvIII overexpressed LN229 cells incubated with unmodified PPVN, antibody-Cy3 (anti-EGFRvIII linked with Cy3) and anti-EGFRvIII modified PPVN-A respectively.

**Figure 3:**
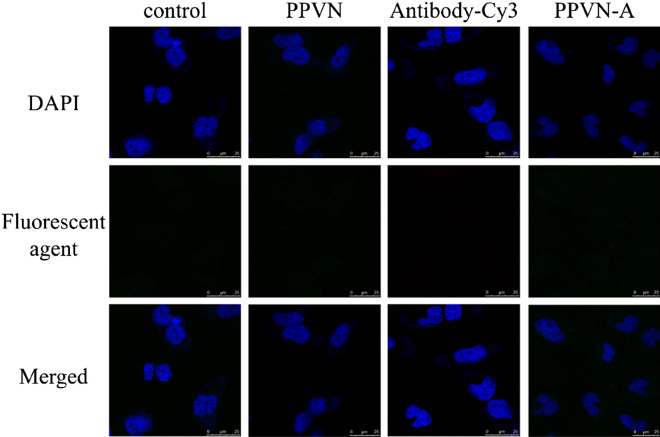
The CLSM of EGFRvIII-negative LN229 cells incubated with unmodified PPVN, antibody-Cy3 (anti-EGFRvIII linked with Cy3) and anti-EGFRvIII modified PPVN-A respectively.

In order to directly visualize the interaction between PPVN-A and EGFRvIII overexpressed or EGFRvIII-negative LN229 cells, targeted imaging was studied by fluorescence microscopy and confocal laser scanning microscopy (CLSM). As displayed in Fig. 2, PPVN-A specifically interacts with EGFRvIII overexpressed LN229 cells which exhibit green fluorescence. In the control experiment, EGFRvIII overexpressed LN229 cells treated with unmodified PPVN were not stained by green fluorescence. Furthermore, the cells cultured with Cy3-labelled anti-EGFRvIII (antibody-Cy3) were detected red fluorescence of Cy3, indicating the selectively binding of anti-EGFRvIII to EGFRvIII overexpressed LN229 cells. To EGFRvIII-negative LN229 cells, as shown in Fig. 3, bare PPVN, antibody-Cy3 and PPVN-A could not be internalized owing to the lack of EGFRvIII receptor on the cells because the corresponding fluorescence has not been detected. The fluorescence microscopy characterization also clearly demonstrates the same results. (Figure S1 and S2) Except for the direct visualization, flow cytometry could be used to quantitate the uptake of nanoparticles which has been reported in previous literature^30^. Moreover, another EGFRvIII overexpressed cell line (U251) was also established and treated with PPVN-A to evaluate the targeting ability. As expected, the fluorescence of PPVN-A was observed on EGFRvIII overexpressed U251 cells (Figure S3), indicating the specificity of PPVN-A toward EGFRvIII.

In order to exclude the effect of the toxicity of PPVN solution on cells, the maximum safe concentration of PPVN should be first determined before photodynamic therapy. We use the method of multiple dilution to prepare different concentrations of PPVN, and then use CCK-8, that could be reduced by dehydrogenase in mitochondria of live cells to produce water-soluble yellow formazan and the amount of formazan is in direct proportion to the number of live cells, to perform cytotoxicity test. At last, it is proved that 7.5 μg/mL was the maximum safe concentration (Figure S4).

**Figure 4:**
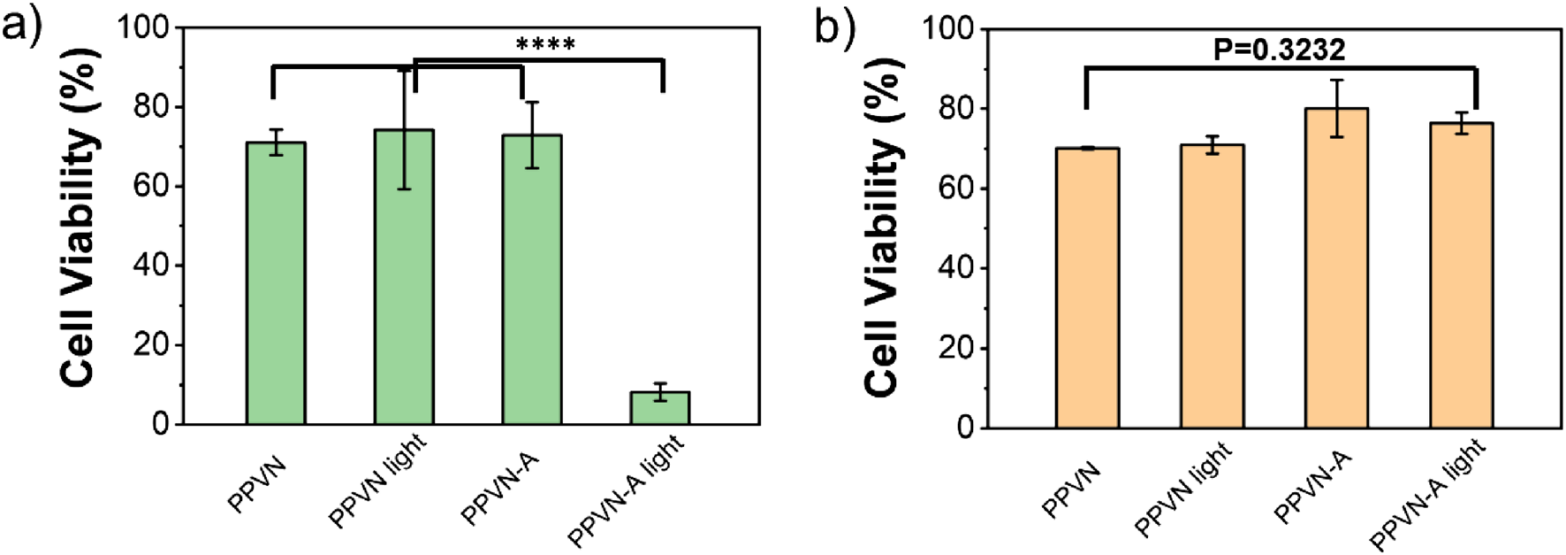
The cell viability of (**a**) EGFRvIII overexpressed LN229 and (**b**) EGFRvIII negative LN229 incubated with PPVN and PPVN-A at the concentration of 5 μg/mL respectively in the absence and presence of white light. ****p < 0.0001.

Based on the ROS generation sensitized by PPVN and the specific binding of PPVN-A to EGFRvIII overexpressed LN229, the targeted antitumor activity of PPVN-A was evaluated by utilizing the CCK method. As displayed in Fig. 4a, PPVN without any modification treated EGFRvIII overexpressed LN229 cells in the presence and absence of light irradiation shows more than 80% cell viability indicating relatively good biocompatibility. Anti-EGFRvIII antibody labelled PPVN-A exhibits tumor cell killing efficiency under the irradiation of white light due to the generation of ROS sensitized by PPV, while the EGFRvIII overexpressed LN229 cells cultured with PPVN-A in the absence of light luminescence retain high viability ratio. EGFRvIII negative LN229 treated with PPVN and PPVN-A under light irradiation or in dark display high cell viability because PPVN-A cannot target EGFRvIII negative cells with the lack of expression of EGFRvIII receptor. (Fig. 4b) The results demonstrate that PPVN-A possesses excellent targeting antitumor activity toward EGFRvIII overexpressed Human Glioma Cells.

In addition, subcutaneous tumor-forming mice were used to verify the photodynamic therapy effect of nanoparticles. BCLB/C nude female mice were subcutaneously injected EGFRvIII overexpressed LN229 cells to establish a tumor-bearing mouse model. The tumor-bearing mice were treated with different conditions through local injection with the aid of white light. The results show that the tumors treated with nanoparticles grow slower than that of control groups. (Figure S6).

## Conclusion

Surface modification on fluorescent conjugated nanoparticles (PPVN) with the antibody of EGFRvIII which is the most important acceptor of glioblastoma cells when treating glioblastoma is achieved. PPVN-A can effectively target glioma cells for specific imaging and photodynamic therapy. On the one hand, the fluorescent targeting effect can enable neurosurgeons to clearly identify the tumor boundary during the neurosurgical operation for more accurately resection of the tumor. On the other hand, the reactive oxygen species released after the light irradiation can not only kill the targeted EGFRvIII positive tumor cells, it also has a certain killing effect on the surrounding non-EGFRvIII positive cells. This kind of targeting nanoparticles may become another powerful weapon in the diagnosis and treatment of gliomas.

## Materials and measurements

MEH-PPV (120,000 Da) were purchased from ADS Dyes, Inc. UV–Vis absorption spectra were taken on a JASCO V-550 spectrophotometer. Fluorescence spectra were measured on a Hitachi F-4500 fluorometer equipped with a xenon lamp excitation source. Zeta potentials were measured on a Nano ZS (ZEN3600) system.

### Methods

#### Preparation and characterization of PPVN

Mix 400 μL of MEH-PPV solution (0.5 mg/mL, THF) with 200 μL of PSMA solution (2 mg/mL, THF), and dilute to 5 mL of THF solution. Then, add 10 mL of double distilled ultra-pure H_2_O (DD H_2_O) under sonication. Keep sonication for 3–5 min to obtain a uniform and transparent liquid. Then, nitrogen was blown into the solution (60 min) to remove THF. Subsequently, the solution was concentrated to 5 mL under continuous nitrogen bubbling in an oil bath at 100 °C. After standing to cool to ambient temperature (26 °C), the solution was filtered through a 0.22 μm filter to obtain the final CPNPs dispersion solution whose concentration was calculated to be 0.04 mg/mL.

### Establishment of EGFRvIII overexpressed glioma cell line

#### Construction of plvx-EGFRvIII plasmid

The EGFRvIII gene was added to the multi cloning site (MCS) of plvx-puro plasmid by ligase to prepare plvx-EGRvIII plasmid. The sequence information and map of plvx-puro plasmid are provided in the Supplementary Materials.

#### Packaging of recombinant lentivirus plvx-EGFRvIII

The plvx-EGFRvIII plasmids and the lentiviral packaging plasmids were co-transfected into 293 T cells. Mix Roche HP transfection reagent and total plasmids in the ratio of 2.5 (μL): 1 (μL). And transfect 20 μg of all kinds of plasmids into 100 mMol 293 T cells. A total of 4 petri dishes were transfected. Virus supernatants were collected at 48 and 72 h after transfection, respectively. A total of approximately 100 mL of virus fluid was collected. Centrifuge to remove cell debris, filter with 0.45 μg syringe filter, perform ultracentrifugation at 100,000 times acceleration of gravity for 2 h, resuspend the virus in PBS buffer, divide into four 500 μL recombinant lentiviral cryotubes, and store in a − 80 °C refrigerator.

#### Titration of recombinant lentivirus plvx-EGFRvIII

The virus titer was determined by qPCR. The virus titer was determined using Real-time PCR to detect the copy number of virus genome integrated in virus-infected cells. The 293 T cells were seeded on a 6-well cell culture plate at a density of 2 × 10^5^ cells/well. After 24 h, they were re-counted. The average number of cells was 5.6 × 10^5^ cells/well. Add 0.5 mL of 100-fold diluted virus solution (10 μl of lentivirus diluted to 1 mL medium) to a six-well plate, add polybrene to 8 ug/mL, remove the virus solution after 24 h, and add 0.5 mL fresh medium containing 10 U/mL DNase I, incubate at 37 °C for 15 min, then replace with fresh medium (2 mL/well), and continue culturing for 48 h. TaqMan real-time PCR detects the copy number of viral WPRE elements and cellular RNase P, respectively. Two copies of RNase P represent one cell, and one copy of WPRE represents the provirus of an integrated lentiviral vector.

#### Lentivirus titer calculation

Through the standard curve, the WPRE copy number and RNase P copy number were calculated as 5279.368 and 21,671.367.

Lentivirus titer (Integration Units/ml, IU/ml) can be calculated by the following formula:

$${\text{Lentivirus titer}} = { }\frac{C \times N \times D}{V}$$

$$C = \frac{5279.368}{{21671.367/2}} = 0.4872$$

Among them, C is relative copy number of lentivirus.; N is Number of cells at the time of transfection (5.6 × 10^5^); D means Dilution Ratio (100-fold dilution); V means volume of medium (0.5 mL).

After calculation, Lentivirus titer (IU/ml) is 5.46 × 10^7^ IU/ml.

### Establishment of EGFRvIII stable cell line (LN229 cell line)

Adherent cells (LN229) were plated into a 6-well plate at 1 × 10^6^ cells/well. After 18 to 24 h, ensure that the number of cells is approximately 2 × 10^6^. Replace the original medium with 1 mL medium containing 6 μg/mL Polybrene, and incubate at 37 °C; add 2 mL fresh culture medium after 4 h to diluted Polybrene, continue culturing for 24 h, replacing the original medium with fresh medium.

### Verification of EGFRvIII stable cell line

Western Blot immunoblotting and immunofluorescence were used to verify the stable EGFRvIII cell line.

### Preparation of Anti-EGFRvIII modified PPVN (PPVN-A)

① Configure 5 mg/mL EDCI solution, 21.7% NHS solution and 5% PEG solution for use.

② Activated carboxyl group: add the prepared NPCPs solution (0.04 mg/mL) into a brown light-proof vial, then add 80 μL of EDCI solution and 40 μL of NHS solution, and place on a shaker for 30 min (room temperature).

③ Link EGFRvIII antibody: add 40 μL HEPES buffer, 40 μL PEG solution (5%) and 20 μL EGFRvIII antibody to the above solution, and continue to react on the shaker for 4 h (room temperature).

④ Ultrafiltration: The above solution was ultrafiltered using a 100 KD ultrafiltration tube. After removing the filtered solution, 200 μL of HEPES buffer was used to dissolve the nanoparticles to obtain PPVN-A solution.

### Photodynamic anticancer experiment

Use CCK-8 to test the effect of cell photodynamic therapy, and calculate the cell survival rate and inhibition rate under PPVN-A treatment. The specific steps are as follows:

Inoculate the cell suspension (3000 cells/well) in a 96-well plate, place the culture plate in the incubator for 24 h. Add a safe concentration of nanoparticles and DMEM to the culture plate Mixed solution of culture medium; incubate the culture plate in a 37 °C incubator for 1 h; remove the medium and wash twice with PBS. Irradiate with an illuminator for 10 min with an irradiation intensity of 100 mW/cm^2^. Then, incubate for 1 h again, and remove the medium and wash twice with PBS. Add 10 μL of CCK-8 solution, place the culture plate in the incubator for 1 to 4 h; measure the absorbance at 450 nm using a microplate reader.

### Animal experiment

#### Establish a tumor-bearing mouse model

BCLB/C nude female mice (average body weight: 20.2 g) were subcutaneously injected EGFRvIII overexpressed LN229 cells to establish a tumor-bearing mouse model (Injection volume: 2 × 10^6^ cells/200 μL) Tumor-bearing mouse model establishment finished in 10 days.

#### Photodynamic antitumor therapy

The tumor-bearing mice were divided into 4 groups with 10 mice in each group. In the treatment group, the mouse tumor was injected with drugs (100 μL) and light for 15 min at the fluence of 100 mW/cm^2^ every other day. Measurement of tumor cell diameter and calculation of volume were conducted daily. After 25 days treatment, mice were executed and tumors were taken out for measurement.

#### Live subject statement

The animal part was carried out in compliance with the ARRIVE guidelines in the lab of the Institutional Animal Care and Use Committee of Beijing Vital River Laboratory Animal Technology Co., Ltd. All experimental protocols were approved by Institutional Animal Care and Use Committee of Beijing Vital River Laboratory Animal Technology Co., Ltd. The permission code is P2019008. The animal species used in this study is the mice. All methods were performed in accordance with the relevant guidelines and regulations.

## Supplementary Information

Supplementary Information
