# Supplementary material for: Poly(p-phenylenevinylene) nanoparticles modified with antiEGFRvIII for specific glioblastoma therapy
Source: Sci Rep. 2021 Feb 24;11:4449. doi: 10.1038/s41598-021-83931-5 (PMC7904835; doi:10.1038/s41598-021-83931-5)
Supplement: Supplementary file 1 — Supplementary Information [file 41598_2021_83931_MOESM1_ESM.docx]

Supplementary Materials

Poly(*p*‑phenylenevinylene) nanoparticles modified with antiEGFRvIII for specific glioblastoma therapy

***Yuchao Liang ^1^, Zelin Li ^2^, Huanxiang Yuan ^2,^*, Lei Wang ^1,^* and Li-Hua Gao ^2,^****

*^1^ Neurosurgery Department, Beijing Tian Tan Hospital, Capital Medical University, Beijing 100050, P. R. China; Dingo811@163.com (Y. L.), wanglei_tiantan@163.com (L. W.)*

*^2^ Department of Chemistry, College of Chemistry and Materials Engineering, Beijing Technology and Business University, Beijing 100048, P. R. China; 18811651833@163.com (Z. L.), yhx@iccas.ac.cn (H. Y.), gaolh@th.btbu.edu.cn (L. G.)*

******* *Correspondence: yhx@iccas.ac.cn (H. Y.), wanglei_tiantan@163.com (L. W.), gaolh@th.btbu.edu.cn (L. G.)*

Sequence information：Homo sapiens epidermal growth factor receptor transcript variant III (EGFRvIII).

>ATGCGACCCTCCGGGACGGCCGGGGCAGCGCTCCTGGCGCTGCTGGCTGCGCTCTGCCCGGCGAGTCGGGCTCTGGAGGAAAAGAAAGGTAATTATGTGGTGACAGATCACGGCTCGTGCGTCCGAGCCTGTGGGGCCGACAGCTATGAGATGGAGGAAGACGGCGTCCGCAAGTGTAAGAAGTGCGAAGGGCCTTGCCGCAAAGTGTGTAACGGAATAGGTATTGGTGAATTTAAAGACTCACTCTCCATAAATGCTACGAATATTAAACACTTCAAAAACTGCACCTCCATCAGTGGCGATCTCCACATCCTGCCGGTGGCATTTAGGGGTGACTCCTTCACACATACTCCTCCTCTGGATCCACAGGAACTGGATATTCTGAAAACCGTAAAGGAAATCACAGGGTTTTTGCTGATTCAGGCTTGGCCTGAAAACAGGACGGACCTCCATGCCTTTGAGAACCTAGAAATCATACGCGGCAGGACCAAGCAACATGGTCAGTTTTCTCTTGCAGTCGTCAGCCTGAACATAACATCCTTGGGATTACGCTCCCTCAAGGAGATAAGTGATGGAGATGTGATAATTTCAGGAAACAAAAATTTGTGCTATGCAAATACAATAAACTGGAAAAAACTGTTTGGGACCTCCGGTCAGAAAACCAAAATTATAAGCAACAGAGGTGAAAACAGCTGCAAGGCCACAGGCCAGGTCTGCCATGCCTTGTGCTCCCCCGAGGGCTGCTGGGGCCCGGAGCCCAGGGACTGCGTCTCTTGCCGGAATGTCAGCCGAGGCAGGGAATGCGTGGACAAGTGCAACCTTCTGGAGGGTGAGCCAAGGGAGTTTGTGGAGAACTCTGAGTGCATACAGTGCCACCCAGAGTGCCTGCCTCAGGCCATGAACATCACCTGCACAGGACGGGGACCAGACAACTGTATCCAGTGTGCCCACTACATTGACGGCCCCCACTGCGTCAAGACCTGCCCGGCAGGAGTCATGGGAGAAAACAACACCCTGGTCTGGAAGTACGCAGACGCCGGCCATGTGTGCCACCTGTGCCATCCAAACTGCACCTACGGATGCACTGGGCCAGGTCTTGAAGGCTGTCCAACGAATGGGCCTAAGATCCCGTCCATCGCCACTGGGATGGTGGGGGCCCTCCTCTTGCTGCTGGTGGTGGCCCTGGGGATCGGCCTCTTCATGCGAAGGCGCCACATCGTTCGGAAGCGCACGCTGCGGAGGCTGCTGCAGGAGAGGGAGCTTGTGGAGCCTCTTACACCCAGTGGAGAAGCTCCCAACCAAGCTCTCTTGAGGATCTTGAAGGAAACTGAATTCAAAAAGATCAAAGTGCTGGGCTCCGGTGCGTTCGGCACGGTGTATAAGGGACTCTGGATCCCAGAAGGTGAGAAAGTTAAAATTCCCGTCGCTATCAAGGAATTAAGAGAAGCAACATCTCCGAAAGCCAACAAGGAAATCCTCGATGAAGCCTACGTGATGGCCAGCGTGGACAACCCCCACGTGTGCCGCCTGCTGGGCATCTGCCTCACCTCCACCGTGCAGCTCATCACGCAGCTCATGCCCTTCGGCTGCCTCCTGGACTATGTCCGGGAACACAAAGACAATATTGGCTCCCAGTACCTGCTCAACTGGTGTGTGCAGATCGCAAAGGGCATGAACTACTTGGAGGACCGTCGCTTGGTGCACCGCGACCTGGCAGCCAGGAACGTACTGGTGAAAACACCGCAGCATGTCAAGATCACAGATTTTGGGCTGGCCAAACTGCTGGGTGCGGAAGAGAAAGAATACCATGCAGAAGGAGGCAAAGTGCCTATCAAGTGGATGGCATTGGAATCAATTTTACACAGAATCTATACCCACCAGAGTGATGTCTGGAGCTACGGGGTGACTGTTTGGGAGTTGATGACCTTTGGATCCAAGCCATATGACGGAATCCCTGCCAGCGAGATCTCCTCCATCCTGGAGAAAGGAGAACGCCTCCCTCAGCCACCCATATGTACCATCGATGTCTACATGATCATGGTCAAGTGCTGGATGATAGACGCAGATAGTCGCCCAAAGTTCCGTGAGTTGATCATCGAATTCTCCAAAATGGCCCGAGACCCCCAGCGCTACCTTGTCATTCAGGGGGATGAAAGAATGCATTTGCCAAGTCCTACAGACTCCAACTTCTACCGTGCCCTGATGGATGAAGAAGACATGGACGACGTGGTGGATGCCGACGAGTACCTCATCCCACAGCAGGGCTTCTTCAGCAGCCCCTCCACGTCACGGACTCCCCTCCTGAGCTCTCTGAGTGCAACCAGCAACAATTCCACCGTGGCTTGCATTGATAGAAATGGGCTGCAAAGCTGTCCCATCAAGGAAGACAGCTTCTTGCAGCGATACAGCTCAGACCCCACAGGCGCCTTGACTGAGGACAGCATAGACGACACCTTCCTCCCAGTGCCTGAATACATAAACCAGTCCGTTCCCAAAAGGCCCGCTGGCTCTGTGCAGAATCCTGTCTATCACAATCAGCCTCTGAACCCCGCGCCCAGCAGAGACCCACACTACCAGGACCCCCACAGCACTGCAGTGGGCAACCCCGAGTATCTCAACACTGTCCAGCCCACCTGTGTCAACAGCACATTCGACAGCCCTGCCCACTGGGCCCAGAAAGGCAGCCACCAAATTAGCCTGGACAACCCTGACTACCAGCAGGACTTCTTTCCCAAGGAAGCCAAGCCAAATGGCATCTTTAAGGGCTCCACAGCTGAAAATGCAGAATACCTAAGGGTCGCGCCACAAAGCAGTGAATTTATTGGAGCATGA

The map of plvx-puro plasmid:


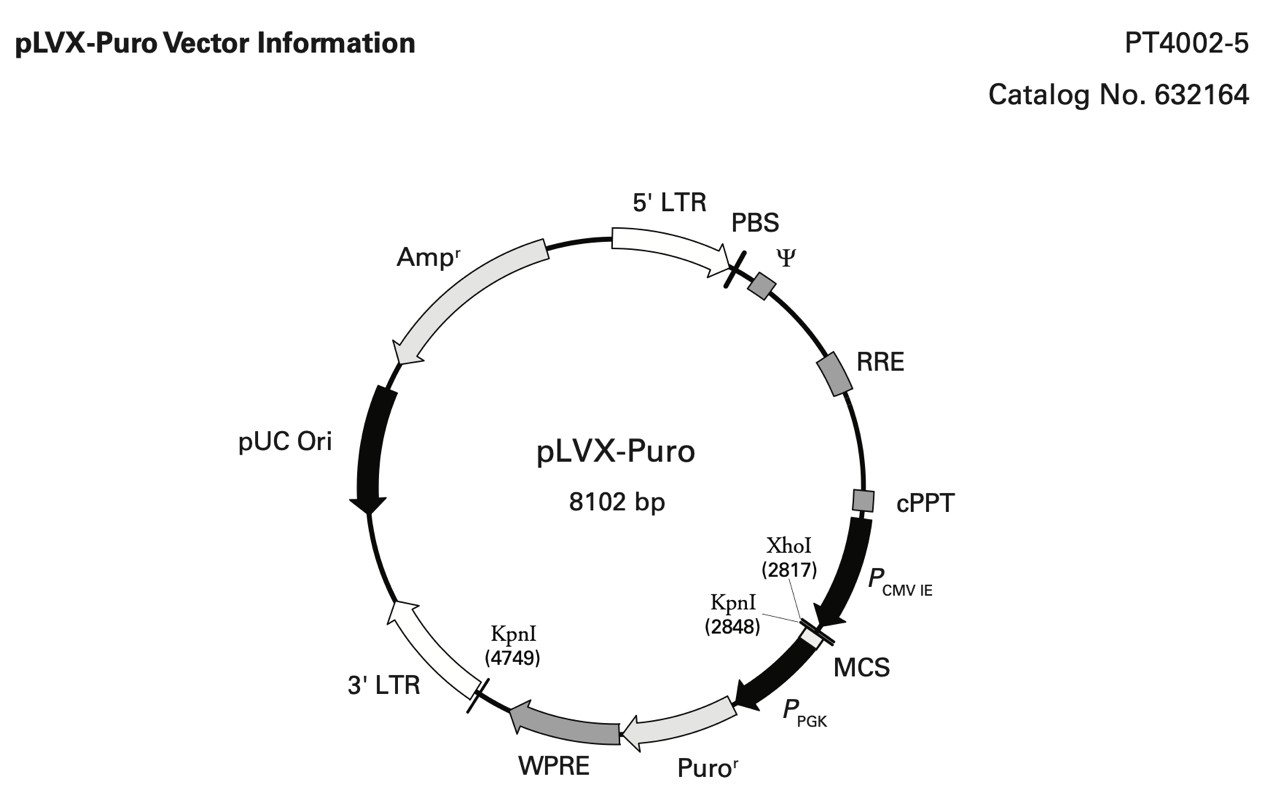


Table S1 The zeta potentials and sizes of PPVN and PPVN-A.


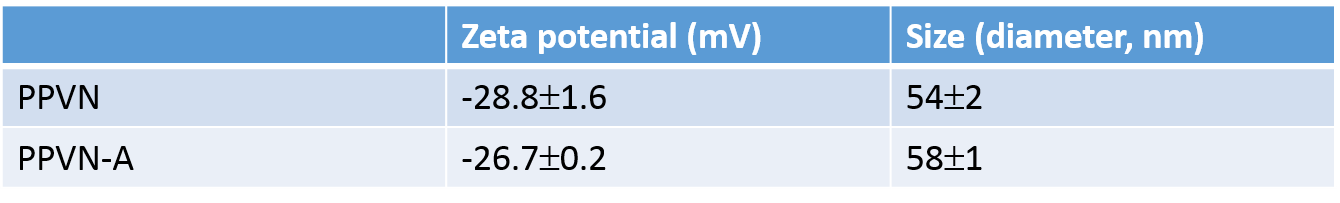


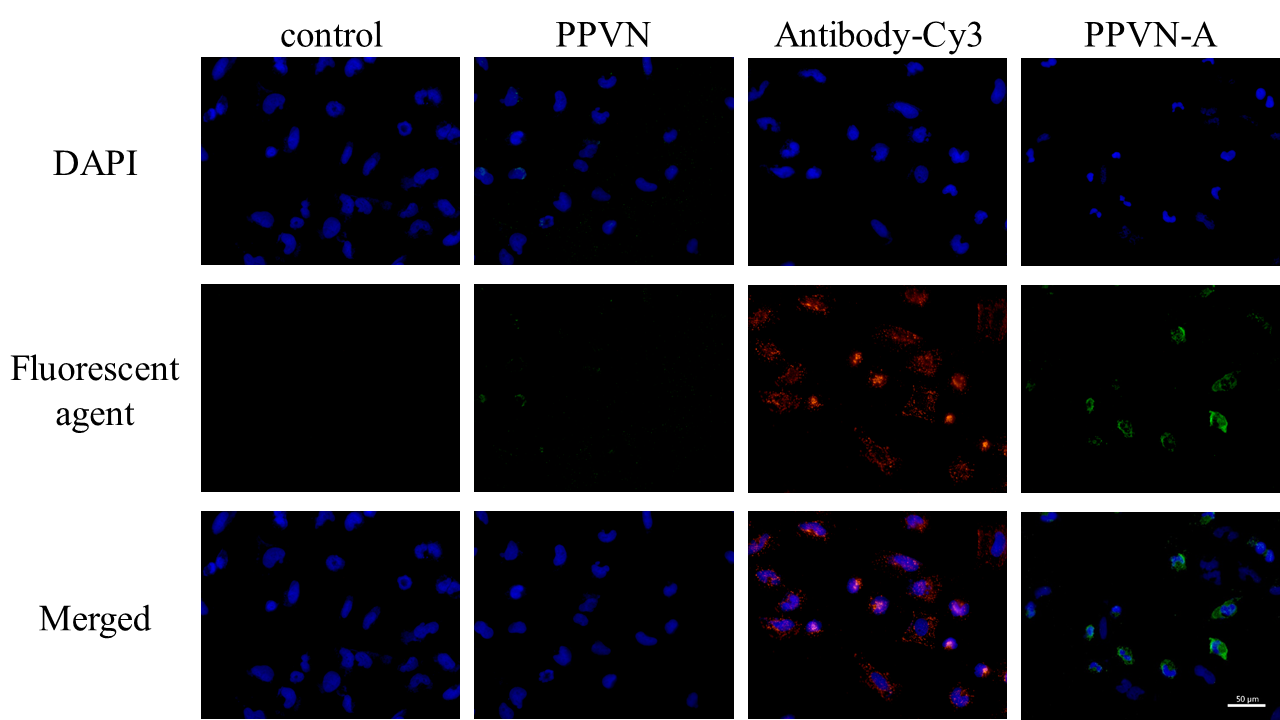


Figure S1 The fluorescence microscopy images of EGFRvIII overexpressed LN229 cells incubated with unmodified PPVN, antibody-Cy3 (anti-EGFRvIII linked with Cy3) and anti-EGFRvIII modified PPVN-A respectively.


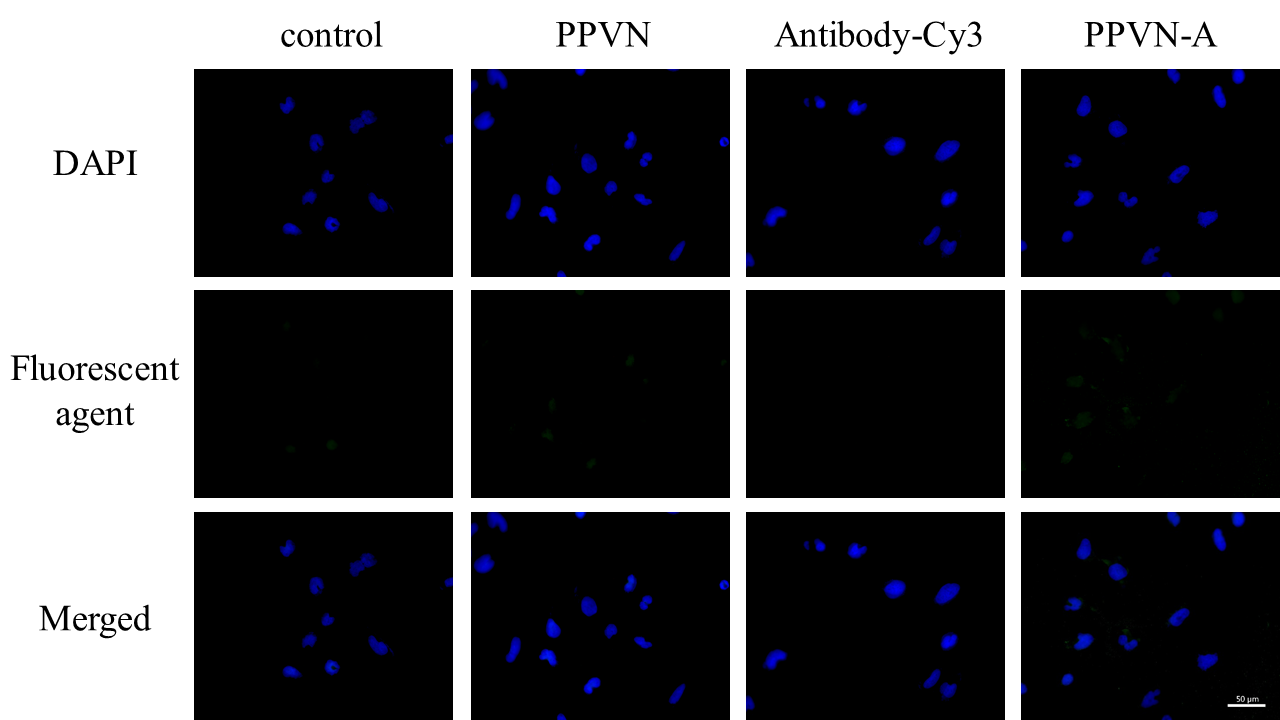


Figure S2 The fluorescence microscopy images of EGFRvIII-negative LN229 cells incubated with unmodified PPVN, antibody-Cy3 (anti-EGFRvIII linked with Cy3) and anti-EGFRvIII modified PPVN-A respectively.


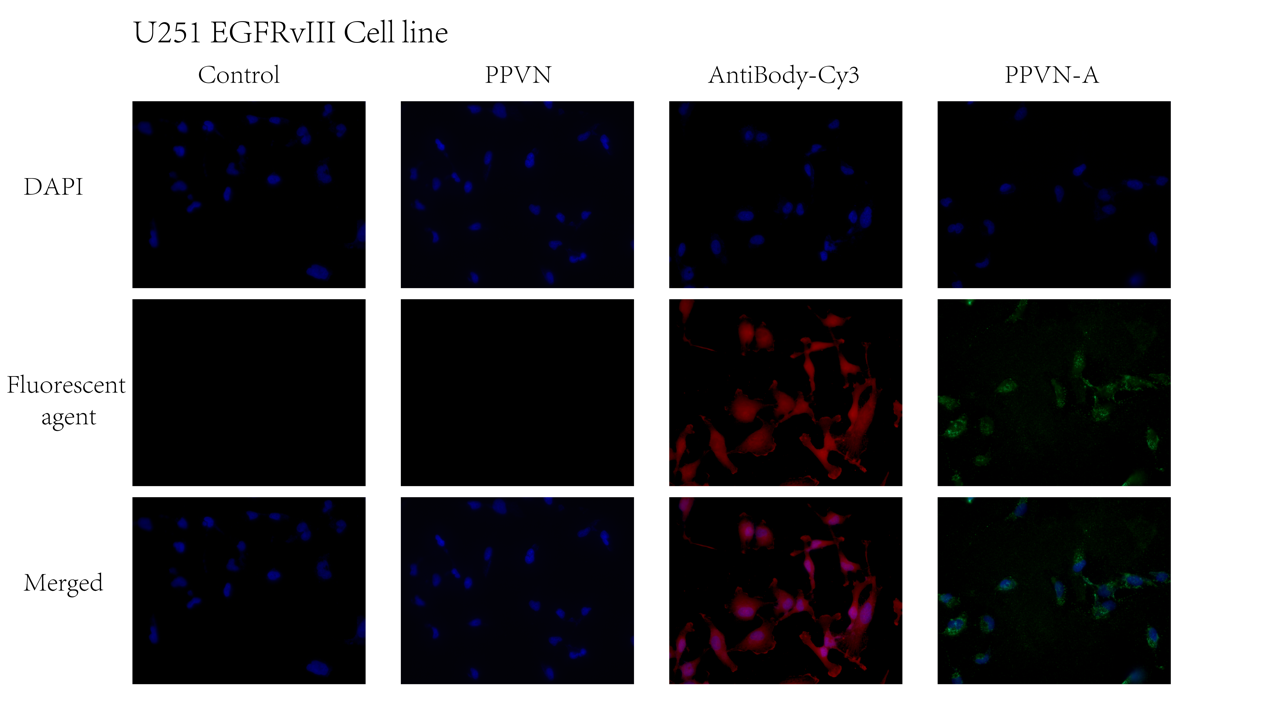


Figure S3 The fluorescence microscopy images of EGFRvIII-overexpressed U251 cells incubated with unmodified PPVN, antibody-Cy3 (anti-EGFRvIII labelled with Cy3) and anti-EGFRvIII modified PPVN-A respectively.


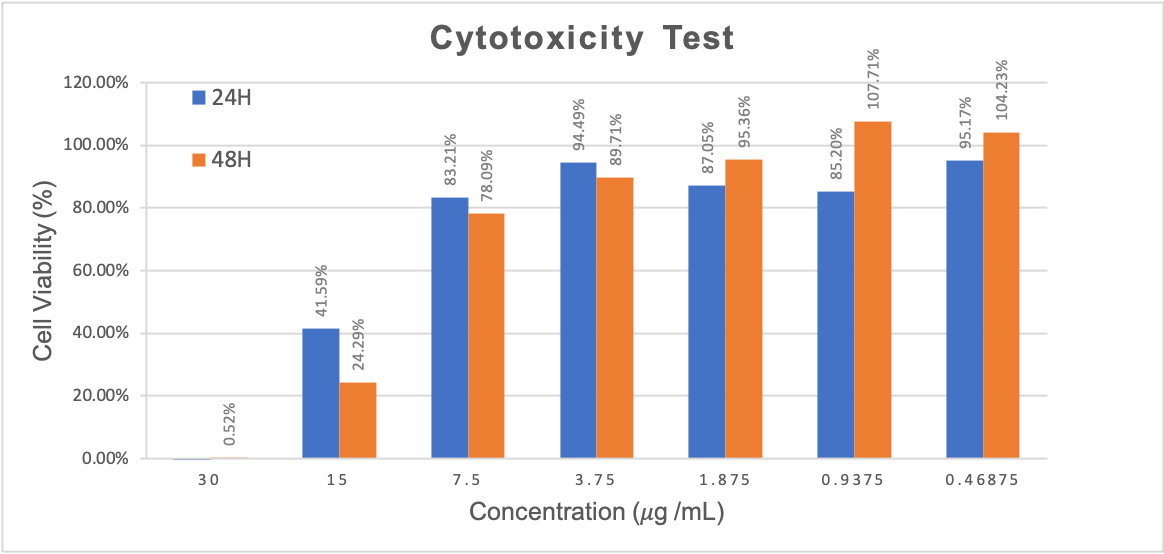


Figure S4 The cell viability of LN229 incubated with different concentrations (𝜇g/mL) of PPVN for 24 h and 48 h using a standard CCK-8 method. 7.5 𝜇g/mL was selected as the max safety concentration.


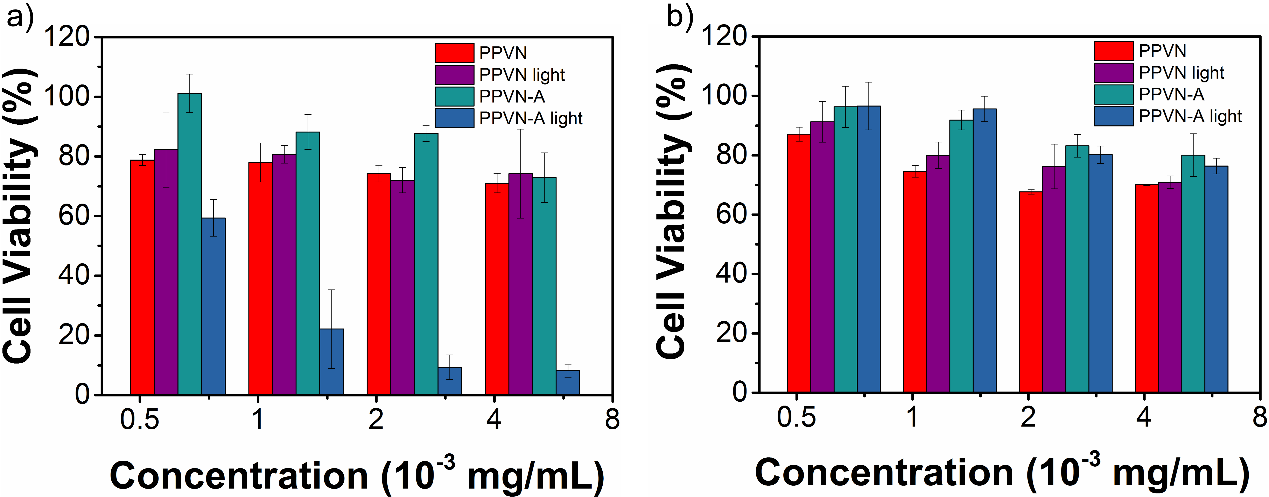


Figure S5 The cell viability of (a) EGFRvIII overexpressed LN229 and (b) EGFRvIII negative LN229 incubated with PPVN and PPVN-A at the concentration of 0.625, 1.25, 2.5, and 5 μg/mL respectively in the absence and presence of white light.


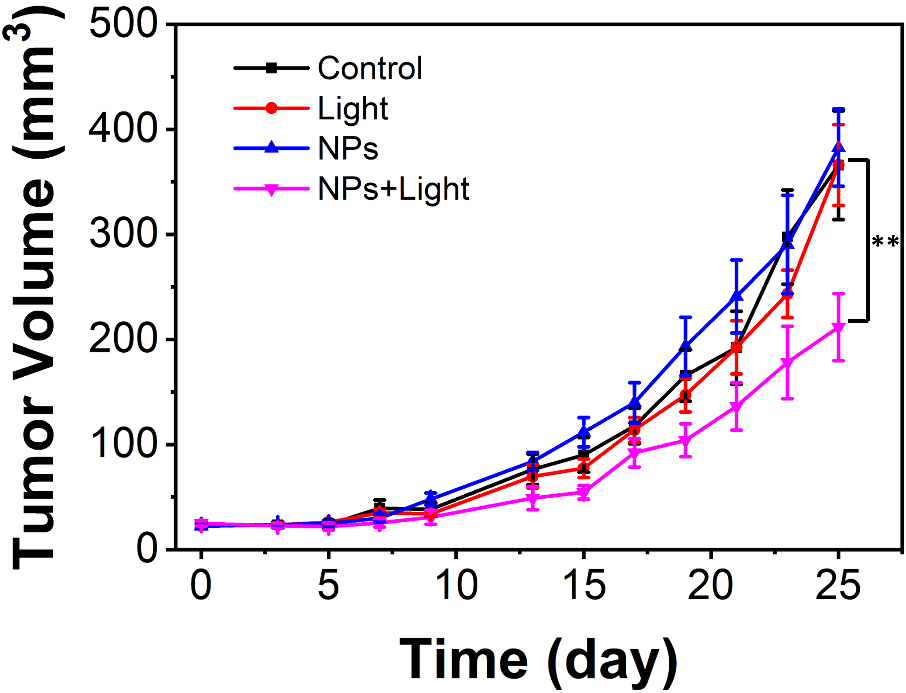


Figure S6 Tumor volume as a function of treatment time. Values are expressed as means ± SD. The value of P < 0.05 of saline treatment vs treatment with NPs.





Figure S7. Zeta potential distribution of PPVN and PPVN-A.
